# Effect of contrast dose, post-contrast acquisition time, myocardial regionality, cardiac cycle and gender on dynamic-equilibrium CMR measurement of myocardial extracellular volume

**DOI:** 10.1186/1532-429X-15-S1-M3

**Published:** 2013-01-30

**Authors:** Christopher A Miller, Josephine H Naish, Glyn Coutts, David Clark, Sha Zhao, Simon G Ray, Mark P Ainslie, Rahul Potluri, Geoffrey Parker, Matthias Schmitt

**Affiliations:** 1 North West Heart Centre, University Hospital of South Manchester, Manchester, UK; 2 Biomedical Imaging Institute, University of Manchester, Manchester, UK; 3 Christie Medical Physics and Engineering, The Christie Hospital, Manchester, UK; 4 Alliance Cardiac MRI Unit, University Hospital of South Manchester, Manchester, UK

## Background

CMR techniques are increasingly being used to evaluate myocardial extracellular volume (ECV). In the most commonly applied method, ECV is quantified using haematocrit-adjusted myocardial and blood T1 values measured before and after gadolinium bolus. The technique is based on a two-compartment model, assuming contrast kinetic effects to be negligible due to a dynamic equilibrium between blood and myocardium (Dynamic-Equilibrium CMR; DynEq-CMR). This study aimed to assess the effect of contrast dose, post-contrast acquisition time, myocardial regionality, cardiac cycle and gender on DynEq-CMR ECV measurement.

## Methods

30 healthy volunteers (asymptomatic, no cardiovascular risk factors, normal examination and ECG; also CMR confirmed normal global and regional function with no LGE in all) were prospectively split into 3 age and sex-matched groups: group A received 0.10mmol/kg Gd-DTPA, group B 0.15mmol/kg and group C 0.20mmol/kg (Table 1). Mid-ventricular short-axis modified look locker inversion recovery (MOLLI) imaging was performed at 1.5T before, and at 2min intervals between 2-20mins after, contrast administration, with same-day hematocrit measurement. MOLLI imaging was repeated in early systole (150ms after R wave) pre- and at 10mins post-contrast. Resulting pixelwise T1 maps (MatLab) were used to calculate ECV. (Phantom studies performed prior to patient scanning determined T1 measurement accuracy and heart-rate correction algorithm).

**Table 1:** Characteristics of healthy volunteers.

	Overall (n=30)	Group A (0.10 mmol/Kg) (n=10)	Group B (0.15 mmol/Kg) (n=10)	Group C (0.20 mmol/Kg) (n=10)	p value
Male	15	5	5	5	
Age	45±13 (22-65	44±14 (23-65)	45±14 (22-64)	46±13 (28-64)	0.98
Age Male	45±15 (22-65)	44±15 (27-65)	46±17 (22-64)	44±15 (30-60)	
Age Female	45±12 (23-64)	45±15 (23-62)	44±12 (28-59)	47±12 (28-64)	
Weight (Kg)	73.1±13.8	74.8±12.9	70.3±16.0	73.1±13.8	0.75
Height (m)	1.69±0.10	1.71±0.09	1.67±0.10	1.70±0.11	0.67
BSA (m2)	1.83±0.20	1.87±0.19	1.78±2.4	1.85±0.18	0.65
Systolic BP (mmHg)	114±11	116±11	114±11	112±12	0.63
Diastolic BP (mmHg)	67±11	71±11	65±13	66±7	0.41
eGFR (mL/min/m2)	95±17	94±11	100±20	91±19	0.50
Indexed EDV (mL/m2)	79±8	78±8	79±7	80±8	0.83
Indexed ESV (mL/m2)	26±5	27±5	27±5	26±5	0.78
EF (%)	67±5	66±4	66±4	68±5	0.45
Indexed Mass (g/m2)	45±8	44±9	43±8	47±7	0.46
HR (bpm)	68±9	67±9	70±11	66±8	0.67

Values are mean ± standard deviation. Age range is also given in brackets. BP indicates blood pressure, eGFR estimated glomerular filtration rate, EDV end diastolic volume; ESV end-systolic volume; EF ejection fraction. ‘Indexed' refers to indexed to body surface area.

## Results

**Figure 1:**
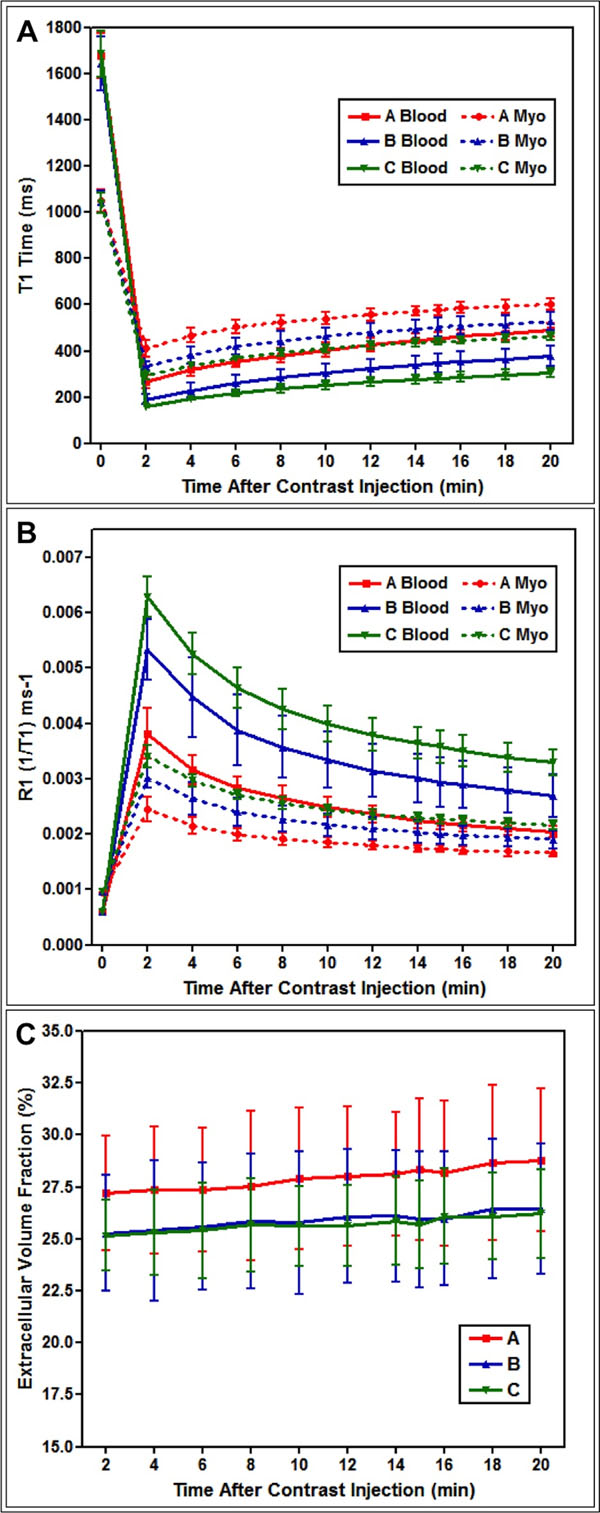
(A) Myocardial (Myo, dashed lines) and blood (continuous lines) T1 relaxation times plotted against time after contrast (Gd-DTPA) administration. Group A (red) received 0.10mmol/kg contrast, Group B (blue) received 0.15mmol/kg and Group C (green) received 0.20mmol/kg. Pre-contrast (Time 0) myocardial and blood T1 relaxation times were not significantly different between groups. Post-contrast T1 relaxation times shortened significantly as contrast dose increased. (B) Myocardial and blood R1 (1/T1) values plotted against time after contrast administration. Labeling as per A. (C) Dynamic-equilibrium CMR-measured myocardial extracellular volume fraction (ECV) plotted against time following contrast administration. ECV was significantly higher in group A compared to group B and group C, but there was no significant difference between groups B and C. ECV increased linearly over time in each group. Error bars represent ±1 SD.

Pre-contrast myocardial (A 1051±49ms; B 1045±49ms; C 1040±43ms; p=0.87) and blood (A 1678±98ms; B 1645±118ms; C 1686±101ms; p=0.66) T1 times did not differ significantly between groups. Mean myocardial (A 542±65ms; B 465±69ms; C 407±55ms; p<0.001) and blood (A 407±73ms; B 307±67ms; C 252±48ms; p<0.001) T1 averaged over all time points post-contrast shortened significantly as contrast dose increased (Figure 1). Mean ECV was significantly higher in group A compared to groups B and C (A 27.7±3.7%; B 25.8±3.4%; C 25.8±2.8%; p<0.001). The difference between groups B and C was not significant. ECV increased linearly over time in each group; between 2 and 20mins post-contrast, ECV increased from 27.2±2.7% to 28.8±3.4%, p=0.020 in group A; 25.3±2.8% to 26.5±3.2%, p=0.004 in group B; and 25.2±1.7% to 26.2±2.1%, p=0.068 in group C. ECV varied significantly between myocardial regions, being highest in the septum and lowest in the lateral wall in each group. ECV did not differ significantly between diastole and systole. ECV was significantly higher in females in each group (A female 29.6±3.0%, male 25.4±3.0%, p<0.001; B female 27.4±2.7%, male 23.6±2.9%, p<0.001; C female 26.1±2.8%, male 24.7±2.5%, p=0.027).

## Conclusions

The small increase in ECV over time suggests that an incomplete dynamic equilibrium between blood and myocardium is achieved. DynEq-CMR-derived ECV varies according to contrast dose, myocardial region and gender.

## Funding

Christopher Miller is supported by a Doctoral Research Fellowship from the National Institute for Health Research (UK).

